# Categorization of nano-structured titanium dioxide according to physicochemical characteristics and pulmonary toxicity

**DOI:** 10.1016/j.toxrep.2016.05.005

**Published:** 2016-05-20

**Authors:** Naoki Hashizume, Yutaka Oshima, Makoto Nakai, Toshio Kobayashi, Takeshi Sasaki, Kenji Kawaguchi, Kazumasa Honda, Masashi Gamo, Kazuhiro Yamamoto, Yasuhiro Tsubokura, Shozo Ajimi, Yoshiyuki Inoue, Nobuya Imatanaka

**Affiliations:** a Chemicals Evaluation and Research Institute, Kurume, Kurume-shi, Fukuoka, Japan; b Chemicals Evaluation and Research Institute, Hita, Hita-shi, Oita, Japan; c Chemicals Evaluation and Research Institute, Japan, Chemicals Assessment and Research Center, Bunkyo-ku, Tokyo, Japan; d National Institute of Advanced Industrial Science and Technology (AIST), Tsukuba, Ibaraki, Japan

**Keywords:** Nano materials, Titanium dioxide, Intratracheal administration, Pulmonary toxicity, Risk assessment

## Abstract

A potentially useful means of predicting the pulmonary risk posed by new forms of nano-structured titanium dioxide (nano-TiO_2_) is to use the associations between the physicochemical properties and pulmonary toxicity of characterized forms of TiO_2_. In the present study, we conducted intratracheal administration studies in rats to clarify the associations between the physicochemical characteristics of seven characterized forms of TiO_2_ and their acute or subacute pulmonary inflammatory toxicity. Examination of the associations between the physicochemical characteristics of the TiO_2_ and the pulmonary inflammatory responses they induced revealed (1) that differences in the crystallinity or shape of the TiO_2_ particles were not associated with the acute pulmonary inflammatory response; (2) that particle size was associated with the acute pulmonary inflammatory response; and (3) that TiO_2_ particles coated with Al(OH)_3_ induced a greater pulmonary inflammatory response than did non-coated particles. We separated the seven TiO_2_ into two groups: a group containing the six TiO_2_ with no surface coating and a group containing the one TiO_2_ with a surface coating. Intratracheal administration to rats of TiO_2_ from the first group (i.e., non-coated TiO_2_) induced only acute pulmonary inflammatory responses, and within this group, the acute pulmonary inflammatory response was equivalent when the particle size was the same, regardless of crystallinity or shape. In contrast, intratracheal administration to rats of the TiO_2_ from the second group (i.e., the coated TiO_2_) induced a more severe, subacute pulmonary inflammatory response compared with that produced by the non-coated TiO_2_. Since alteration of the pulmonary inflammatory response by surface treatment may depend on the coating material used, the pulmonary toxicities of coated TiO_2_ need to be further evaluated. Overall, the present results demonstrate that physicochemical properties may be useful for predicting the pulmonary risk posed by new nano-TiO_2_ materials.

## 1 Introduction

The European Commission defines a nanomaterial as “a natural, incidental or manufactured material containing particles, in an unbound state or as an aggregate or as an agglomerate and where, for 50% or more of the particles in the number size distribution, one or more external dimensions is in the size range 1 nm–100 nm” [1]. Due to this small particle size, the surface area per unit mass of nanomaterials is greater than that of the corresponding bulk materials, and it is this characteristic that gives nanomaterials their unique properties.

Nanomaterials are predicted to soon become the cornerstone of the microelectronics, materials, textiles, energy, healthcare, and cosmetics industries [2]. Indeed, nano-structured titanium dioxide (nano-TiO_2_) is one of the most widely used nanomaterials in the world, and the production volume of nano-TiO_2_, which is increasing annually, is expected to reach nearly 2.5 million metric tons per year in 2025 [3]. Traditionally, TiO_2_ fine particles have been considered to have low toxicity; however, concerns have been raised recently regarding the potential health risks posed by nano-TiO_2_ to consumers, workers, and the environment [4]. In particular, a recently published toxicological review showed that inhalation was the primary route of nano-TiO_2_ exposure and thus highlighted the need for more information on the safety of nano-TiO_2_ [5]. Many researchers and regulators are now trying to understand the hazards of nano-TiO_2_ and determine appropriate strategies for the assessment of the pulmonary risk associated with exposure to nano-TiO_2_ materials. Nano-TiO_2_ can be manufactured in various forms that have different physicochemical characteristics (e.g., crystallinity, shape, particle size, surface area, and surface modification), which can lead to nanomaterials with the same chemical formula but different pulmonary toxicities [6]. Therefore, from a regulatory standpoint, it would be beneficial to assess the pulmonary risk of all newly developed nano-TiO_2_ materials; however, a program of this size is unrealistic due to the time and money that would be required.

One potential means of predicting the pulmonary risk of new forms of nano-TiO_2_ is to use the associations between the physicochemical properties and pulmonary toxicity of characterized forms of TiO_2_. Several researchers have already investigated the relationships between the physicochemical characteristics of different forms of nano-TiO_2_ and their pulmonary toxicities in rats, including the relationship between pulmonary toxicity and particle crystallinity [7], particle size [8], [9], [10], [11], [12], particle surface area [13], [10], [12], and particle surface modification [14], [15], [16]. For example, smaller TiO_2_ particles have been shown to induce a greater acute inflammatory response compared with larger TiO_2_ particles after intratracheal administration in rats [8], [9], [11]. However, Warheit et al. [12] have also shown that the acute inflammatory and cell-injury effects of three TiO_2_ materials with different particle sizes did not differ. Furthermore, in most of these previous studies only two or three test materials were examined and the studies were conducted under different test conditions, thus hampering comparison of the results.

In the present study, to clarify the associations between the physicochemical characteristics of TiO_2_-based materials and their pulmonary toxicity, intratracheal administration studies were conducted in rats by using seven different characterized TiO_2_. To allow a quantitative and statistical approach to be used, all physicochemical characterizations and in vivo studies were performed in the same laboratory under the same test conditions.

## 2 Materials and methods

### 2.1 Test materials and preparation of administration formulations

Seven forms of TiO_2_ were selected for inclusion in this study: AMT-100, MT-150AW, and MP-100 (Tayca Co. Ltd., Japan); TTO-S-3, TTO-S-3 (Coated), and FTL-100 (Ishihara Sangyo Kaisha, LTD., Japan); and P25 (Evonik Industries, Germany). These materials were chosen to allow examination of as many different physicochemical characteristics as possible, i.e., three different types of crystallinity, three different particle shapes, surface coating (untreated or Al[OH]_3_ coating), and a range of different particle sizes (Table 1). Representative scanning electron microscope images (S-4800, Hitachi High-Technologies Co., Japan) of each test material are shown in Fig. S1.

**Table 1:** Physicochemical characteristics of the seven forms of TiO_2._

Material	Crystallinity^a^	Shape^a^	Primary particle size^a^(nm)	Surface area(m^2^/g)	Surface coating^a^	Particle size^b^(nm)	
						Volume average diameter	Number average diameter
AMT-100	anatase	spherical	6	250–300	No	185(161–198)	68.5(60.6–82.6)
MT-150AW	rutile	spindle	long axis:28.8 short axis:7.6	100–120	No	58.5(52.3–65.8)	28.7(19.5–42.6)
TTO-S-3	rutile	spindle	long axis:50–100 short axis:10–20	102	No	61.9(57.2–64.7)	45.8(41.2–48.9)
TTO-S-3 (Coated)	rutile	spindle	long axis:50–100 short axis:10–20	93.0	Al(OH)_3_ coating	241(202–289)	125(119–133)
P25	rutile/anatase(20/80)	spherical	21	50 ± 15	No	99.2(95.6–101)	73.8(65.5–79.8)
MP-100	rutile	spherical	1000	6	No	531(501–549)	289(89.8–393)
FTL-100	rutile	needle	long axis:1680 short axis:130	12.0	No	−c	−c

a Data from catalog or obtained from the manufacturer.

b Average particle diameter was measured in the three different administration formulations by means of dynamic light scattering (Zetasizer Nano ZS; Malvern Instruments Ltd., UK). Figures in parentheses show the smallest to largest value among the three different concentrations. Intensity size distributions measured by dynamic light scattering was converted into volume and number size distributions using Mie theory [23].

c Particle size was not calculated because dynamic light scattering gives little effective information for needle-shaped particles with a large aspect ratio.

Administration formulations were prepared as described in Refs. [17], [18] (Table S1). Briefly, 2 g of test material was dispersed in 50 mL of 2 mg/mL of disodium phosphate (food additive grade; Wako Pure Chemical Industries, Ltd., Japan), which was prepared by using endotoxin-free pure water. The suspension was sonicated in a glass bottle by using an ultrasonic bath (5510J-MT; Branson Ultrasonics Co., USA) for one to three hours and then centrifuged at 20–1000*g* for 5–40 min at 20 °C (CF16RXII and T15A41; Hitachi Koki Co., Ltd., Japan). The supernatant was collected as a stock suspension. Administration formulations were prepared by diluting the stock suspension to the appropriate concentration with 2 mg/mL disodium phosphate. The concentration of test material in the administration formulations was determined by using a weight analysis method, where the weight loss of the suspension was measured with a balance scale (AUW220D; Shimadzu Co., Japan) after drying at 200 °C in a thermostatic chamber (ON-300S; As One Co., Japan). The particle size and size distribution of the test materials in the administration formulations were measured by means of dynamic light scattering (Zetasizer Nano ZS; Malvern Instruments Ltd., UK). In principle, particle size and size distribution can be expressed in terms of mass, volume, or number of particles [19]. However, since a robust understanding of which means of expression is the best for understanding the pulmonary toxicity of TiO_2_ in rats is yet to be obtained, two different values—volume average diameter and number average diameter—obtained from the dynamic light scattering analysis were used in the present study (Table 1 and Fig. S2). The particle size of FTL-100 was not determined because dynamic light scattering gives little effective information for needle-shaped particles with a large aspect ratio.

### 2.2 Test animals

Male F344/DuCrlCrlj rats were obtained from Charles River Laboratories Japan, Inc. Animals at 12 weeks of age with body weights of 212.3–282.1 g on the day of administration were used in the in vivo studies. The animals were housed in animal rooms equipped with a local barrier system and were maintained at 21–25 °C and 40% to 70% relative humidity with 10–15 air changes per hour and a photoperiod of 12 h of light per day (lights on, 7:00; lights off, 19:00). The study was approved by the Institutional Animal Care and Use Committee prior to the start of the study.

### 2.3 Test conditions

In the in vivo studies, one vehicle control group (2 mg/mL of disodium phosphate aqueous solution without test material) and three treatment groups (0.67, 2, or 6 mg/kg TiO_2_) were used for each test material. The majority of previous studies using intratracheal administration of TiO_2_ in rats were conducted with doses in the range of 0.75–6 mg/kg TiO_2_ [8], [9], [20], [11], [12], [7]. Therefore, based on the results of these previous studies, the doses in the present study were chosen so that toxic effects but not death or severe suffering were induced at the highest dose and so that adverse effects were avoided at the lowest dose. Forty rats were used for each test material (i.e., ten test animals/dose). Test animals were anesthetized by means of isoflurane inhalation and angled approximately 45° on a restraining stand. Administrations were conducted by using a stomach sonde (Natsume Seisakusho Co., Ltd., Japan) or MicroSprayer Aerosolizer (Model IA-1B-R for Rat, Penn-Century, Inc., USA) inserted transorally into the tracheal lumen at a depth of approximately 6 cm from the angle of the mouth. The volumes of the administrations were 1 mL/kg for AMT-100, MT-150AW, TTO-S-3, TTO-S-3 (Coated), P25 and MP-100, and 2 mL/kg for FTL-100, based on the animal’s body weight on the day of administration. To assess the acute and subacute pulmonary toxicity of each test material, bronchoalveolar lavage fluid (BALF) examination and pathological examination were conducted at three days (acute phase) and at four weeks (subacute phase) after intratracheal administration. The examinations were not conducted earlier than three days after administration because the results would likely reflect the initial pulmonary inflammatory response to the bolus administration of liquid into the lungs [8]. On the day of examination, ten test animals per dose were euthanized by means of bleeding via the ventral aorta under pentobarbital anesthesia. Five animals were used for the BALF examination, and five animals were used for the pathological examination. Test animals were fasted for 16–20 h prior to euthanasia and dissection. In all animals, clinical signs were observed daily after intratracheal administration, and body weight was recorded before the administration of the test materials, at three and seven days after administration, and once per week thereafter until the end of the study.

### 2.4 BALF examination

The trachea of each rat was cannulated and 7 mL of saline (Otsuka Pharmaceutical Co., Ltd, Japan) was instilled into the lungs by means of gravity; that is, saline was instilled into the lungs from 30 cm above the test animal and then drained from the lungs to a bottle that was placed 30 cm under the test animal. The lavage procedure was conducted twice (i.e., a total of 14 mL of saline was instilled into the lungs). Approximately 90% of the instilled saline volume was retrieved. Total cell counts were determined in a portion of the BALF by using an ADVIA 120 hematology analyzer (Siemens Healthcare Diagnostics, USA). The remaining BALF was centrifuged (400*g*, 10 min, 4 °C), and the total protein content, concentrations of albumin, lactate dehydrogenase (LDH) activity, and alkaline phosphatase (ALP) activity in the supernatant were determined by using a Hitachi Automatic Clinical Analyzer 7170 (Hitachi High-Technologies Corporation Co., Japan). The cell pellets were re-suspended in phosphate-buffered saline and smeared onto glass slides; 200 cells per specimen were counted to determine the leukocyte differential (neutrophils, macrophages, lymphocytes, basophils, and eosinophils).

### 2.5 Pathological examinations

The lungs, trachea, parathymic lymph nodes, posterior mediastinal lymph node, liver, kidneys, spleen, and brain were removed from the rats. The weights of the lungs, liver, kidneys, spleen, and brain were measured to calculate relative organ weights per body weight (g/100 g). The organs were fixed with 10% (v/v) neutral phosphate-buffered formalin solution, stained with hematoxylin–eosin, and examined histopathologically under a light microscope.

### 2.6 Associations between the physicochemical characteristics of TiO_2_ and the pulmonary inflammatory response in rats

To investigate the associations between the physicochemical characteristics of the TiO_2_ (i.e., crystallinity, shape, particle size, and surface coating) and the pulmonary inflammatory response to the TiO_2_ in rats (i.e., acute or subacute), we used three BALF parameters—differential neutrophil ratio (an indicator of inflammatory response), total protein content (an indicator of permeability of vessels to protein in blood), and LDH activity (an indicator of cell injury). These BALF parameters have previously been used to evaluate the pulmonary toxicity of nano-TiO_2_ in rats [14], [8], [21], [22], [11], [16], [12], [7]. When analysing the dose-dependency of the three BALF parameters, we suspected that at three days after intratracheal administration of the TiO_2_, some of the parameters might be saturated in the rats administered 6 mg/kg TiO_2_. Therefore, the values of the BALF parameters obtained for the rats administered 2 mg/kg TiO_2_ were used. To standardize the results obtained for each test material, the results of the BALF examinations are expressed as the difference between the value in the rats administered 2 mg/kg TiO_2_ and that in the control rats.

### 2.7 Statistical analysis

Statistical analyses of body weight, BALF parameters, and relative organ weight were conducted by using the StatLight software (Yukms Co., Ltd., Japan). Bartlett’s test for homogeneity of variance was conducted, and when the variances were homogeneous at a significance level of 5%, Dunnett’s test was conducted. When the variances were not homogeneous, Dunnett’s test for nonparametric data was conducted. Statistical significances were judged at the 5% probability level. Regression analyses were performed by using Microsoft Office Excel 2013 and GraphPad Prism 6 version 6.01 (GraphPad Software, Inc., USA) for Windows.

## 3 Results

### 3.1 Clinical signs and body weight

No mortalities or abnormalities in clinical signs were observed in either the control or treatment animals for all test materials. The body weight of all rats increased with increasing age, and there were no significant differences in body weight between the control and treatment groups for all test materials (data not shown).

### 3.2 BALF examinations

#### 3.2.1 Cells in BALF

**Fig. 1:**
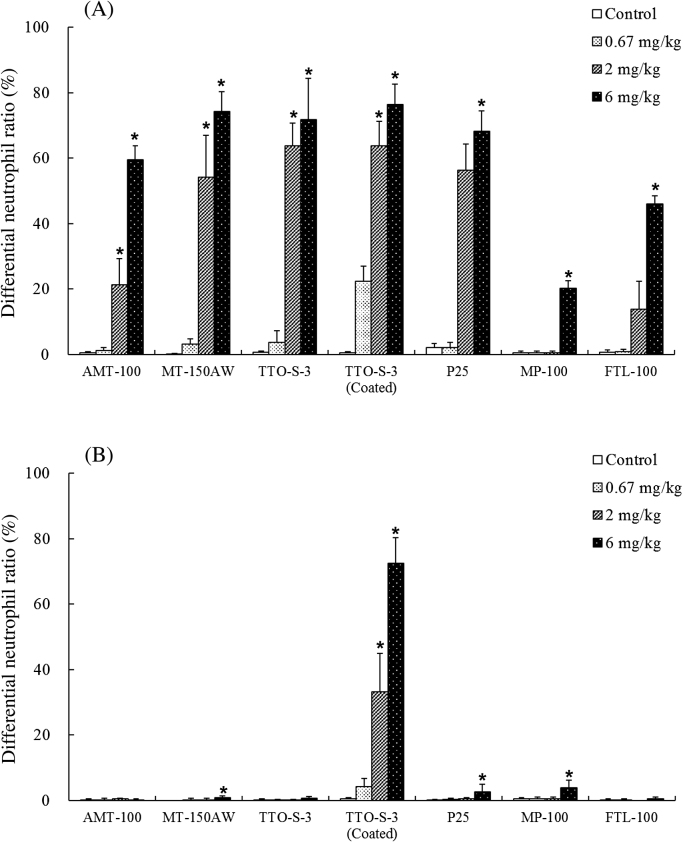
Differential neutrophil ratio in bronchoalveolar lavage fluid at three days (A) and at four weeks (B) after intratracheal administration of various forms of TiO_2_. Values are presented as average ± SD. * significant difference from control group (*P* < 0.05).

The results of the BALF examinations at three days and at four weeks after administration of the test materials are shown in Figs. 1 and S3−6. At three days after administration, dose-dependent increases in the differential neutrophil ratio were observed for all of the test materials (Fig. 1A). At four weeks after administration, although significant increases in differential neutrophil ratio remained in the animals administered 6 mg/kg MT-150AW, P25, or MP-100, the increases in differential neutrophil ratio had recovered to levels comparable with those in the control groups, except for in the animals administered 2 or 6 mg/kg TTO-S-3 (Coated) (Fig. 1B). Similar results were observed for total cell count (Fig. S3), differential macrophage ratio (Fig. S4), and differential lymphocyte ratio (Fig. S5). No significant differences in differential eosinophil ratio at three days or at four weeks after administration were observed for any of the test materials (Fig. S6).

#### 3.2.2 Biochemical examination of BALF

**Fig. 2:**
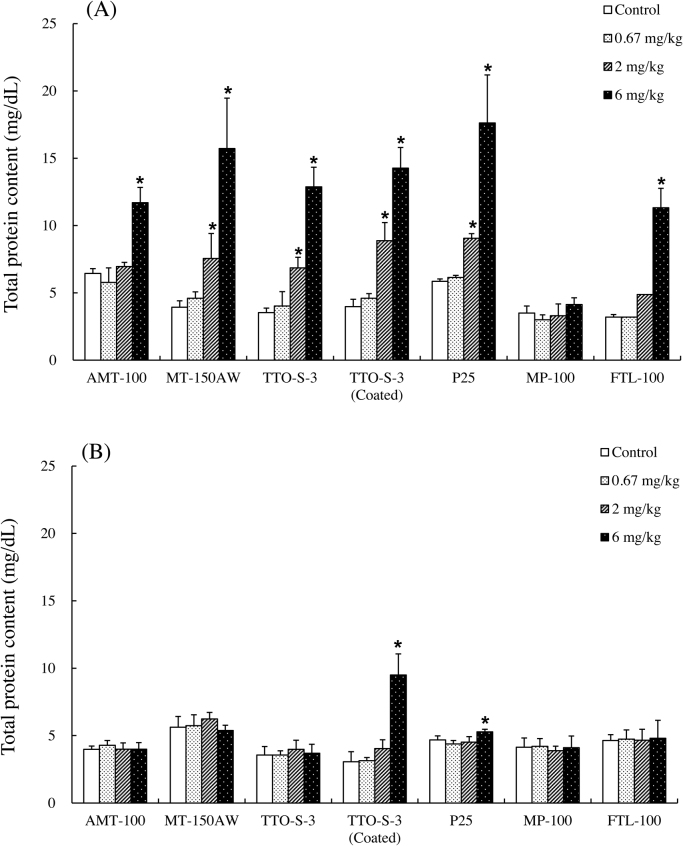
Total protein content in bronchoalveolar lavage fluid at three days (A) and at four weeks (B) after intratracheal administration of various forms of TiO_2_. Values are presented as average ± SD. * significant difference from control group (*P* < 0.05).

**Fig. 3:**
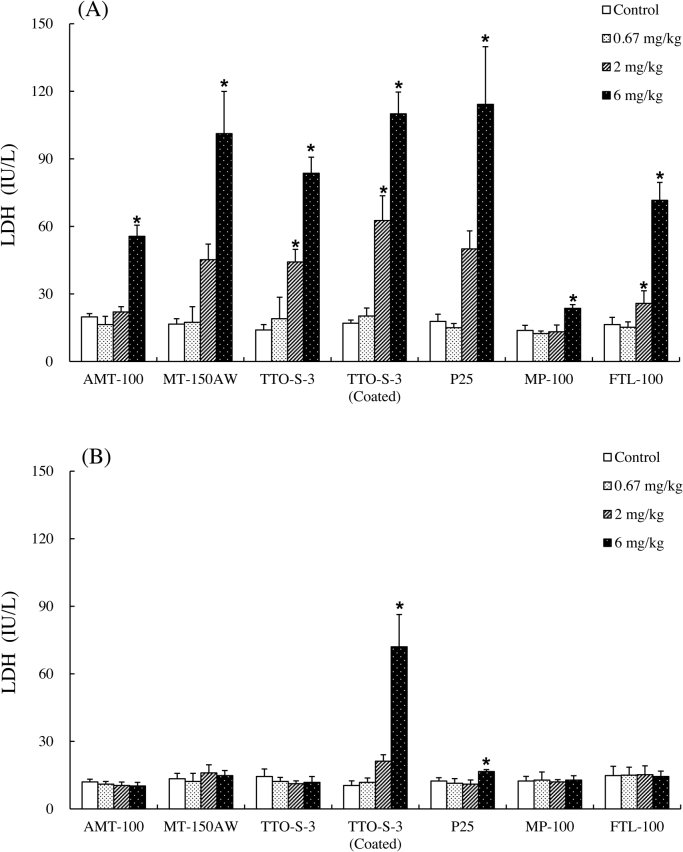
Lactate dehydrogenase (LDH) activity in bronchoalveolar lavage fluid at three days (A) and at four weeks (B) after intratracheal administration of various forms of TiO_2_. Values are presented as average ± SD. * significant difference from control group (*P* < 0.05).

The results of the biochemical examination of BALF are shown in Figs. 2, 3, S7, and S8. At three days after administration, dose-dependent increases in total protein content were observed for all of the test materials except MP-100 (Fig. 2A). At four weeks after administration, although significant increases in total protein content remained in the animals administered 6 mg/kg P25, the increases in total protein content had recovered to levels comparable with those in the control groups for all test materials, except for in the rats administered 6 mg/kg TTO-S-3 (Coated) (Fig. 2B). Similar trends were observed for LDH activity (Fig. 3), albumin concentration (Fig. S7), and ALP activity (Fig. S8).

### 3.3 Pathological examinations

#### 3.3.1 Relative organ weight

**Fig. 4:**
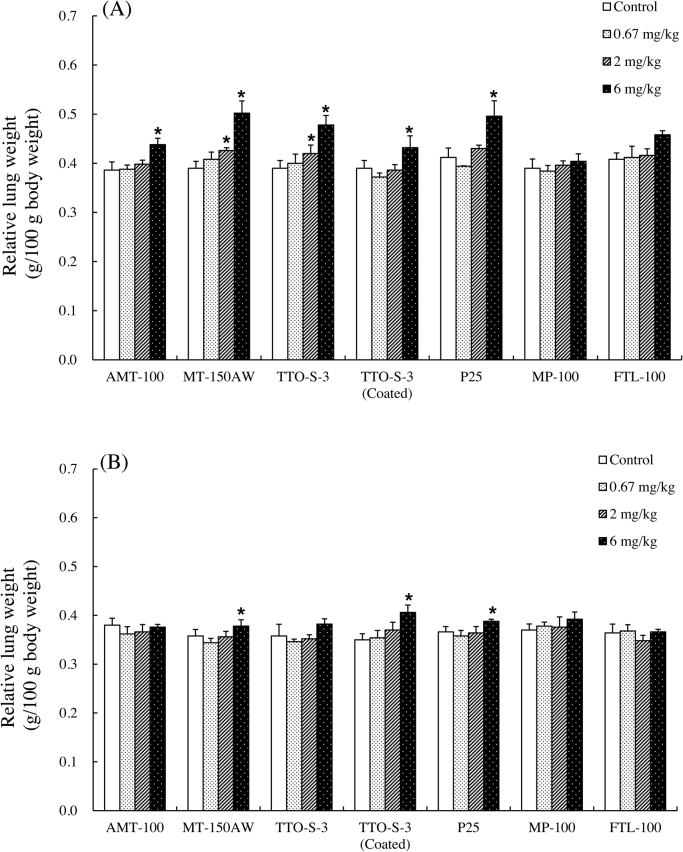
Relative lung weight at three days (A) and at four weeks (B) after intratracheal administration of various forms of TiO_2_. Values are presented as average ± SD. * significant difference from control group (*P* < 0.05).

At three days after administration, significant increases in relative lung weight were observed in the rats administered 6 mg/kg TiO_2_, except in those administered MP-100 or FTL-100 (Fig. 4A). For MT-150AW or TTO-S-3, significant increases were also observed in the animals administered 2 mg/kg. At four weeks after administration, significant increases in relative lung weight remained in the animals administered 6 mg/kg MT-150AW, TTO-S-3 (Coated), or P25 (Fig. 4B). No significant differences were observed in the relative organ weight of liver, kidney, spleen, and brain for any of the test materials (data not shown).

#### 3.3.2 Histopathological examinations

**Fig. 5:**
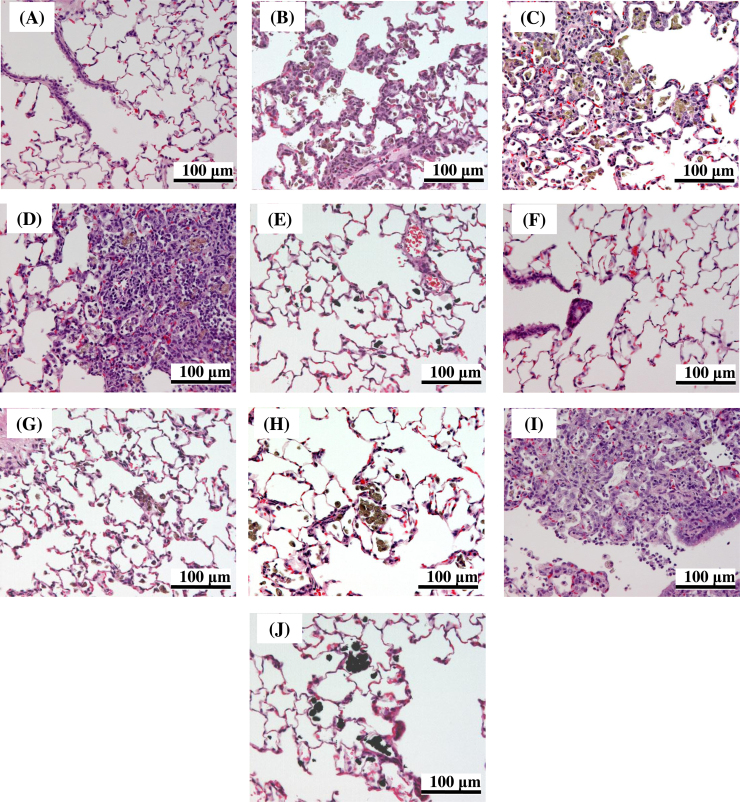
Representative light micrographs of lung tissue from rats after intratracheal administration of 6 mg/kg of the indicated form of TiO_2_. At three days after intratracheal administration: (A) Control, (B) MT-150AW, (C) TTO-S-3, (D) TTO-S-3 (Coated), (E) MP-100. At four weeks after intratracheal administration: (F) Control, (G) MT-150AW, (H) TTO-S-3, (I) TTO-S-3 (Coated), (J) MP-100.

At three days after administration, inflammation in the lung was observed for all test materials. Infiltration of inflammatory cells (neutrophils and alveolar macrophages) or hyperplasia of the alveolar epithelium with thickening of the alveolar wall, or both, were observed for all test materials (Table 2 and Fig. 5). Most of the histopathological changes were very slight with only MT-150AW and P25 inducing mild changes at the highest dose at three days after administration. The frequency and grade of the histopathological changes increased in a dose-dependent manner. At four weeks after administration, the inflammatory responses had subsided in all groups, except for in the animals administered TTO-S-3 (Coated). In the animals administered TTO-S-3 (Coated), infiltration of inflammatory cells (neutrophils and foamy alveolar macrophages) or hyperplasia of the alveolar epithelium with thickening of the alveolar wall were observed (Table 2 and Fig. 5). These findings are consistent with the results of the BALF examinations, in which six of the seven test materials induced acute responses, whereas TTO-S-3 (Coated) induced a subacute response. No toxicological findings were observed in any of the other organs examined for any of the test materials.

**Table 2:** Comparison of frequency and grade of histopathological responses.

Test material																					
	AMT-100			MT-150AW			TTO-S-3			TTO-S-3 (Coated)			P25			MP-100			FTL-100		
Administration dose (mg/kg)																					
	0.67	2	6	0.67	2	6	0.67	2	6	0.67	2	6	0.67	2	6	0.67	2	6	0.67	2	6
(At three days after administration)																					
Infiltration of inflammatory cells (neutrophils and alveolar macrophages)																					
±	1	1	3	0	2	0	1	2	0	0	1	2	0	0	1	1	0	1	1	0	3
+	0	0	1	0	3	5	0	2	5	0	0	3	0	0	4	0	0	0	0	0	1

Hyperplasia of alveolar epithelium																					
±	0	1	0	0	1	0	0	0	1	0	0	0	0	0	0	1	0	1	0	0	2
+	0	0	0	0	0	0	0	0	4	0	0	0	0	0	3	0	0	0	0	0	2
++	0	0	0	0	0	5	0	0	0	0	0	0	0	0	2	0	0	0	0	0	0
(At four weeks after administration)																					
Infiltration of inflammatory cells (neutrophils and foamy alveolar macrophages)																					
±	0	0	0	0	0	0	0	0	0	0	2	0	0	0	0	0	0	0	0	0	0
+	0	0	0	0	0	0	0	0	0	0	0	5	0	0	0	0	0	0	0	0	0

Hyperplasia of alveolar epithelium																					
+	0	0	0	0	0	0	0	0	0	0	0	2	0	0	0	0	0	0	0	0	0

Five animals were observed in each group.

±, very slight change; +, slight change; ++, mild change.

### 3.4 Associations between physicochemical characteristics of TiO_2_ and pulmonary inflammatory responses in rats

#### 3.4.1 Crystallinity

**Fig. 6:**
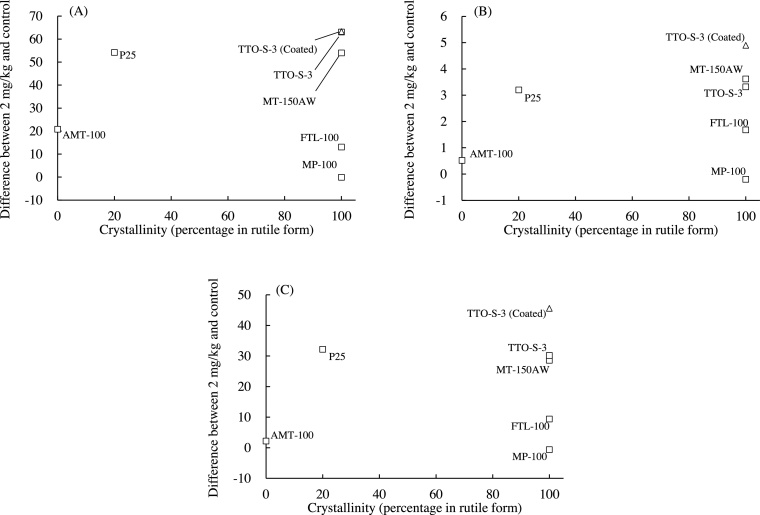
Associations between crystallinity (percentage of TiO_2_ in rutile form) and bronchoalveolar lavage fluid parameter values at three days after intratracheal administration of indicated form of TiO_2_. (A) Differential neutrophil ratio, (B) Total protein content, (C) Lactate dehydrogenase (LDH). Values are presented as the average of the difference between the value for the 2-mg/kg group and that of the control group. Percentage of TiO_2_ in rutile form: AMT-100, 0%; MT-150AW, 100%; TTO-S-3, 100%; TTO-S-3 (Coated), 100%; P25, 20%; MP-100, 100%; FTL-100, 100%.

The associations between the crystallinity of the test materials and the values of three BALF parameters (differential neutrophil ratio, total protein content, and LDH activity) determined at three days after administration are shown in Fig. 6. To examine these associations quantitatively and statistically, the percentage of the materials in rutile form, as determined by the manufacturer, was used as an index of crystallinity (Table 1). For example, the percentage of materials in the rutile form for AMT-100 was 0%, that of MT-150AW was 100%, and that of P25 was 20%. The BALF parameter values for the predominantly rutile materials varied greatly, and no clear trends were observed between the percentage of material in the rutile form and any of the BALF parameter values. Similarly, there were also no clear associations between the percentage of material in the rutile form and the values of the BALF parameters at four weeks after administration (data not shown). Therefore, it is unlikely that the crystallinity of the test materials was associated with the acute pulmonary inflammatory responses observed in the rats.

#### 3.4.2 Shape

**Fig. 7:**
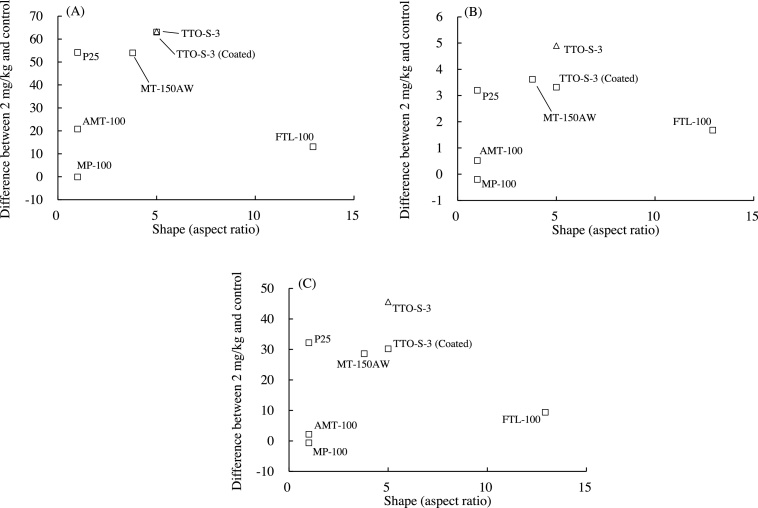
Associations between aspect ratio and bronchoalveolar lavage fluid parameter values three days after intratracheal administration of various forms of TiO_2_. (A) Differential neutrophil ratio, (B) Total protein content, (C) Lactate dehydrogenase (LDH). Values are presented as the average of the difference between the value for the 2-mg/kg group and that of the control group. Aspect ratios: AMT-100, 1.00; MT-150AW, 3.79; TTO-S-3, 5.00; TTO-S-3 (Coated), 5.00; P25, 1.00; MP-100, 1.00; FTL-100, 12.9.

The associations between the shape of the test materials and the values of the three BALF parameters determined at three days after administration are shown in Fig. 7. To examine these associations quantitatively and statistically, the shape of each test material was expressed as its aspect ratio (i.e., long axis/short axis). No clear associations were found between aspect ratio and inflammatory response at three days (Fig. 7) or at four weeks after administration of the test materials (data not shown). Therefore, it is unlikely that particle shape was associated with the acute pulmonary inflammatory responses observed in the rats.

#### 3.4.3 Particle size

**Fig. 8:**
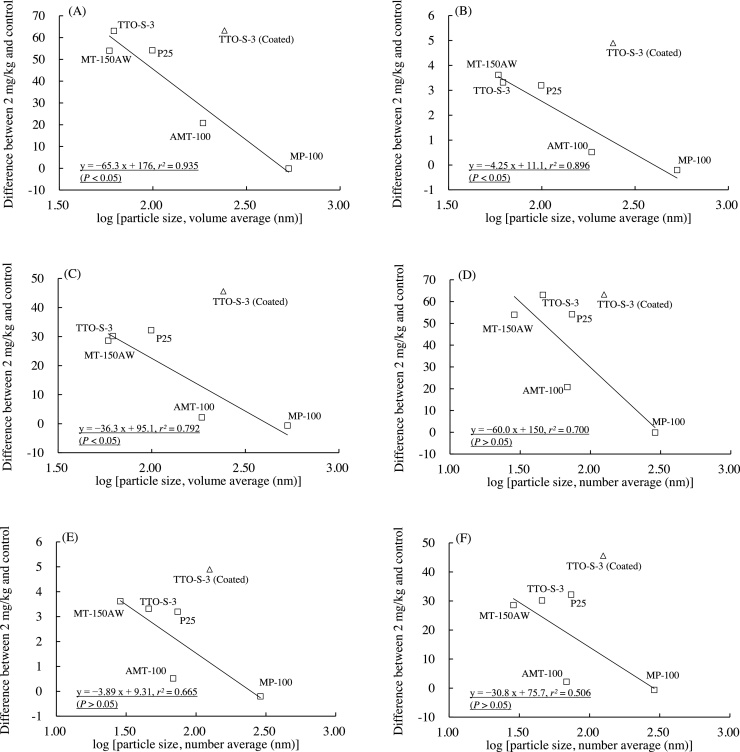
Associations between particle size (nm) and bronchoalveolar lavage fluid parameter values three days after intratracheal administration of various forms of TiO_2_. Volume average diameter: (A) Differential neutrophil ratio, (B) Total protein content, (C) Lactate dehydrogenase (LDH). Number average diameter: (D) Differential neutrophil ratio, (E) Total protein content, (F) LDH. Solid line is the linear regression line derived from five test materials (excluding the data for TTO-S-3 (Coated) and FTL-100). Values are presented as the average of the difference between the value for the 2-mg/kg group and that of the control group.

The associations between the particle size of the test materials and the values of the three BALF parameters determined at three days after administration are shown in Fig. 8. The data for FTL-100 were not plotted because the particle size measured by means of dynamic light scattering would have given little effective information due to the material’s large aspect ratio. In Fig. 8, linear relationships are presented between the logarithm of particle size and BALF parameter value. Strong correlations were observed when the data for TTO-S-3 (Coated) were excluded from the regression analysis expressed by using Eqs. (1)–(6):

(1) $$\text{Differential}\text{neutrophil}\;\text{ratio}=-65.3\times \text{log}\;(\text{volume}\text{average})+176,\;r^{2}=0.935$$

(2) $$\text{Total}\text{protein}\text{content}=-4.25\times \log(\text{volume}\text{average})+11.1,r^{2}=0.896$$

(3) $$\text{LDH}=-36.3\times \log(\text{volume}\text{average})+95.1,r^{2}=0.792$$

(4) $$\text{Differential}\text{neutrophil}\text{ratio}=-60.0\times \log(\text{number}\text{average})+150,r^{2}=0.700$$

(5) $$\text{Total}\text{protein}\text{content}=-3.89\times \log(\text{number}\text{average})+9.31,r^{2}=0.665$$

(6) $$\text{LDH}=-30.8\times \log(\text{number}\text{average})+75.7,r^{2}=0.506$$

The correlations between BALF parameter value and logarithm of particle size at three days after administration were strong for all three BALF parameters examined, that is, the *r^2^* values for volume average diameter were high, and the correlations were statistically significant (*P* < 0.05). No clear associations between BALF parameter value and particle size were observed at four weeks after administration (data not shown). Therefore, it is likely that particle size, especially volume average diameter, was associated with the acute—but not the subacute—pulmonary inflammatory response observed in the rats.

#### 3.4.4 Surface coating

In the plots of BALF parameter value versus particle size at three days after administration, the plot of TTO-S-3 (Coated) was located separate from the plots for the other test materials (Fig. 8A–F). Furthermore, the results of the BALF examinations and histopathological examinations at four weeks after administration showed that TTO-S-3 (Coated) continued to induce an inflammatory response whereas the other test materials did not (Fig. 1, Fig. 2, Fig. 3, Fig. 4, Fig. 5). Therefore, it is likely that the Al(OH)_3_ coating the surface of the particles in TTO-S-3 (Coated) had a marked effect on the pulmonary inflammatory response in rats.

#### 3.4.5 Categorization of seven TiO_2_

**Fig. 9:**
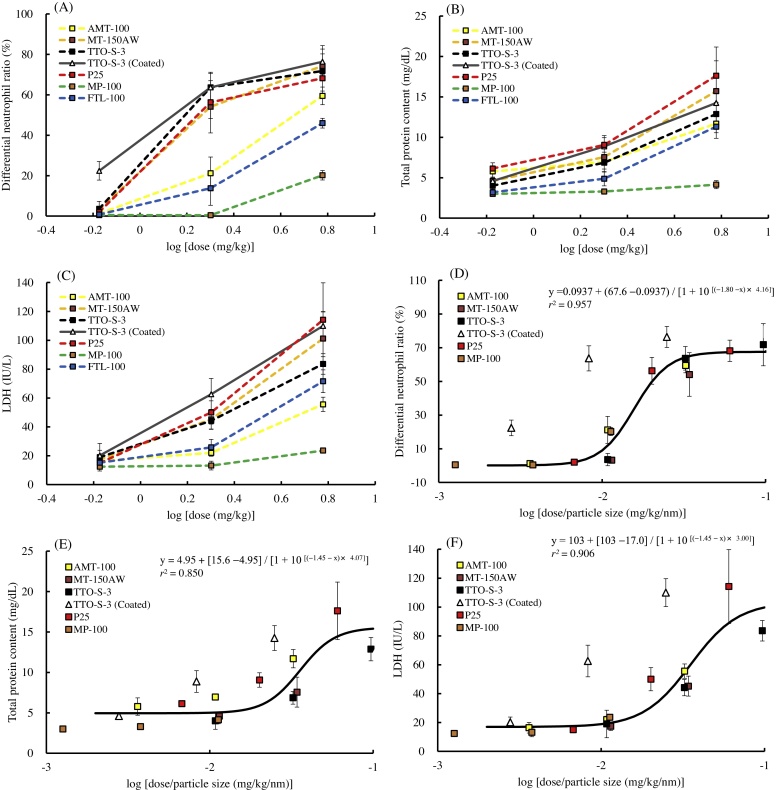
Dose—response curves for bronchoalveolar lavage fluid parameters three days after intratracheal administration of various forms of TiO_2_. Administration doses expressed as mass (mg/kg): (A) Differential neutrophil ratio, (B) Total protein content, (C) Lactate dehydrogenase (LDH). Administration doses normalized by particle size (mg/kg/nm): (D) Differential neutrophil ratio, (E) Total protein content, (F) LDH. Solid lines are the sigmoidal dose—response curve derived from five test materials (excluding the data for TTO-S-3 (Coated) and FTL-100). Values were taken from the 2-mg/kg group and are presented as average ± SD.

Using the present results, we categorized the seven TiO_2_ into two groups: a group containing TiO_2_ with no surface coating (i.e., AMT-100, MT-150AW, TTO-S-3, P25, MP-100, and FTL-100) and a group containing the TiO_2_ with surface coating (i.e., TTO-S-3 [Coated]). The dose–response curves for the BALF parameters (differential neutrophil ratio, total protein content, and LDH activity) at three days after administration are shown in Fig. 9. When the administration doses were expressed as mass (mg/kg), the curves for the BALF parameter values varied greatly (Fig. 9A–C); however, when the administration doses of each test material were normalized by particle size (e.g., volume average), that is, by dividing the mass administration dose (mg/kg) by the particle size (nm) (Fig. 9D–F), the curves for the BALF parameters after administration of the TiO_2_ from the first group of materials (i.e., the materials with no surface coating) all fitted the same sigmoidal dose–response curve (*r^2^* = 0.957 [differential neutrophil ratio], 0.850 [total protein content], 0.906 [LDH]). This suggests that for the first group of TiO_2_, the non-coated TiO_2_, the acute pulmonary inflammatory response (at three days after intratracheal administration) is equivalent when the particle size is the same, regardless of crystallinity or shape.

## 4 Discussion

In the present study, all seven TiO_2_ induced acute pulmonary inflammatory responses at three days after intratracheal administration in rats, which included increases in the differential neutrophil ratio, total protein content, and LDH activity in BALF; infiltration of inflammatory cells in the lung; and the development of hyperplasia of the alveolar epithelium. For six of the seven test materials, these inflammatory responses had recovered at four weeks after administration to levels comparable to those in the control groups, which is consistent with the results of a previous study Nöel et al. [21]. However, rats administered TTO-S-3 (Coated) continued to exhibit inflammatory responses at four weeks after administration, indicating that this TiO_2_ also induced subacute pulmonary toxicity.

The relationships between the physicochemical characteristics of the TiO_2_ and the results of the BALF examination showed that smaller TiO_2_ particles induced a greater acute pulmonary inflammatory response than did larger TiO_2_ particles, irrespective of particle crystallinity or shape (Fig. 8A–8F). Consistent with this result, the frequencies and grades of the histopathological findings (infiltration of inflammatory cells and hyperplasia of the alveolar epithelium) at three days after administration of the test materials were also greater for the smaller TiO_2_ particles than for the larger TiO_2_ particles (Table 2 and Fig. 5). Several previous studies have shown that smaller TiO_2_ particles induce a greater acute inflammatory response than do larger TiO_2_ particles after intratracheal administration [8], [9], [11]. However, Warheit et al. [12] evaluated the acute lung toxicity in rats after intratracheal administration of three TiO_2_ with different particle sizes (primary particle size: approx. 300 nm [particles], 200 nm × 35 nm [rods], and approx. 10 nm [dots]) and showed that there were no differences in the acute inflammatory response and cell injury induced by these three materials. A robust understanding of the effect of TiO_2_ particle size on acute pulmonary toxicity in rats is yet to be obtained; however, in the present study, we characterized and tested seven forms of TiO_2_ in the same laboratory under identical test conditions, and our results suggest that particle size, especially volume average diameter, may be associated with the acute pulmonary inflammatory response to intratracheal administration of TiO_2_ in rats.

Our results also showed that the surface coating of TiO_2_ particles might have a marked effect on the pulmonary inflammatory response to intratracheal administration of TiO_2_ in rats. Several previous studies have shown that surface treatment affects the pulmonary toxicity of TiO_2_ in rats. For example, Oberdörster [15] showed that silane-coated TiO_2_ induces a much lower pulmonary inflammatory response compared with untreated TiO_2_, and Höhr et al. [14] showed that hydrophobic surface treatment of TiO_2_ (achieved through methylation) reduces total cell numbers and induces less influx of neutrophils into BALF compared with untreated TiO_2_. Similarly, Warheit et al. [16] showed that TiO_2_ surface-treated with alumina (Al_2_O_3_) and amorphous silica (SiO_2_) (82% TiO_2_ + 7% Al_2_O_3_ + 11% SiO_2_) induces mild adverse pulmonary effects when compared with the effects induced by untreated TiO_2_. Together with these previous results, the present results indicate that altering the surface structure of TiO_2_ particles affects the rat pulmonary inflammatory response to those particles. Furthermore, the present results also show that the type of pulmonary inflammatory response may be related to the coating material used. That is, in the present study, TTO-S-3 (Coated), which is surface-coated with Al(OH)_3_, induced both an acute and a subacute pulmonary inflammatory response, whereas uncoated TTO-S-3 induced only an acute pulmonary inflammatory response. Further studies are needed to determine the relationships between surface treatments and pulmonary inflammatory response.

It is known that nanomaterial-induced pulmonary inflammation depends largely on the extent of deposition and clearance of the material in the lungs [4], [22]. Our group previously examined the pulmonary clearance kinetics of TiO_2_ in rats by using the same seven test materials assessed in the present study and reported that the pulmonary clearance rate constant of TTO-S-3 (Coated) (0.00018–0.011/day) was much lower than those for the other six uncoated TiO_2_ (0.0073–0.020/day) [18]. Therefore, slower clearance from the lung may account for the subacute pulmonary inflammatory response observed after administration of TTO-S-3 (Coated) in the present study, although further studies are required to elucidate the detailed toxicological mechanism.

Together, the present results allow the following three conclusions to be drawn regarding the relationship between the physicochemical characteristics of nano-TiO_2_ and the pulmonary inflammatory response after intratracheal administration in rats: (1) crystallinity and shape are unlikely to be associated with acute pulmonary inflammatory responses to TiO_2_; (2) particle size is likely associated with the acute pulmonary inflammatory response to TiO_2_; and (3) surface coating has the potential to alter the pulmonary inflammatory response to TiO_2_.

We next categorized the seven TiO_2_ into two groups: a group containing the six TiO_2_ with no surface coating (i.e., AMT-100, MT-150AW, TTO-S-3, P25, MP-100, and FTL-100) and a group containing the one TiO_2_ with a surface coating (i.e., TTO-S-3 [Coated]). Intratracheal administration to rats of the TiO_2_ from the first group of materials induced acute pulmonary inflammatory responses, and within this group, the acute pulmonary inflammatory response (at three days after intratracheal administration) was equivalent when the particle size was the same, regardless of crystallinity or shape. Therefore, the acute pulmonary inflammatory responses in this group may be predicted by using the correlation between the particle size of and inflammatory responses induced by characterized forms of TiO_2_. In contrast, intratracheal administration to rats of the TiO_2_ from the second group (i.e., TTO-S-3 [Coated]) induced a more severe, subacute pulmonary inflammatory response. The pulmonary toxicity of coated TiO_2_ needs to be further evaluated since the alteration of the pulmonary inflammatory response by surface treatment may depend on the coating material used.

In summary, the present study provides a possible means of predicting the pulmonary risk of new nano-TiO_2_ by using the known associations between the physicochemical characteristics and pulmonary toxicity of characterized forms of TiO_2_. Additional studies using TiO_2_ with different surface treatments, and examination of the toxicological mechanisms behind the alteration of the pulmonary inflammatory response by surface treatment, will help in constructing a robust rationale for predicting the pulmonary toxicity of nano-TiO_2_ based on their physicochemical characteristics.

## Conflict of interest

This study was conducted under the “Development of Innovative Methodology for Safety Assessment of Industrial Nanomaterials” supported by the Ministry of Economy, Trade and Industry (METI) of Japan.

## Transparency document

Transparency Document
