# Supplementary material for: Categorization of nano-structured titanium dioxide according to physicochemical characteristics and pulmonary toxicity
Source: Toxicol Rep. 2016 May 20;3:490–500. doi: 10.1016/j.toxrep.2016.05.005 (PMC5615941; doi:10.1016/j.toxrep.2016.05.005)
Supplement: Supplementary file 2 [file mmc1.doc]

**SUPPLEMENTAL DATA**

Categorization of nano-structured titanium dioxide according to physicochemical characteristics and pulmonary toxicity

Naoki Hashizume^a*†^, Yutaka Oshima^b†^, Makoto Nakai^c†^, Toshio Kobayashi^b^, Takeshi Sasaki^d^, Kenji Kawaguchi^d^, Kazumasa Honda^d^, Masashi Gamo^d^, Kazuhiro Yamamoto^d^, Yasuhiro Tsubokura^b^, Shozo Ajimi^b^, Yoshiyuki Inoue^a^, Nobuya Imatanaka^c^

a Chemicals Evaluation and Research Institute, Japan, Kurume, Kurume-shi, Oita, Japan

b Chemicals Evaluation and Research Institute, Japan, Hita, Hita-shi, Oita, Japan

c Chemicals Evaluation and Research Institute, Japan, Chemicals Assessment and Research Center, Bunkyo-ku, Tokyo, Japan

d National Institute of Advanced Industrial Science and Technology (AIST), Tsukuba, Ibaraki, Japan

† The first three authors equally contributed to the manuscript.

*Corresponding author:

Naoki Hashizume

Chemicals Evaluation and Research Institute, Kurume, Kurume-shi, Fukuoka, Japan

Telephone: +81 (942) 34-1500

Fax: +81 (942) 39-6804

[hashizume-naoki@ceri.jp](mailto:hashizume-naoki@ceri.jp)

Table S1. Preparation methods for administration formulations.

|  | Ultra sonication  (h) | Centrifugal acceleration  (×g) | Centrifugal time  (min) |
| --- | --- | --- | --- |
| AMT-100 | 1 | 350 | 5 |
| MT-150AW | 3 | 1000 | 25 |
| TTO-S-3 | 3 | 1000 | 30 |
| TTO-S-3 (Coated) | 3 | 100 | 40 |
| P25 | 3 | 1000 | 20 |
| MP-100 | 1 | 20 | 15 |
| FTL-100 | 3 | 100 | 12 |

| AMT-100  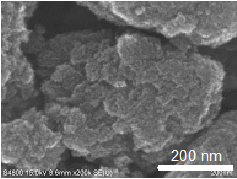 | MT-150AW  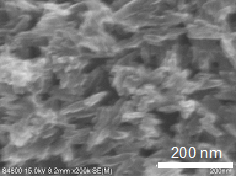 | TTO-S-3  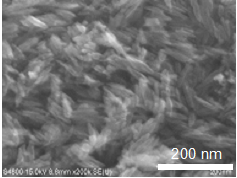 |
| --- | --- | --- |
| TTO-S-3 (Coated)  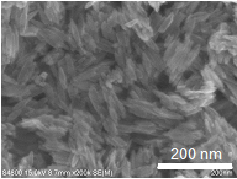 | P25  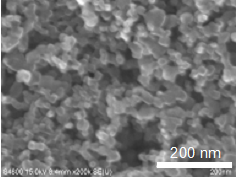 | MP-100  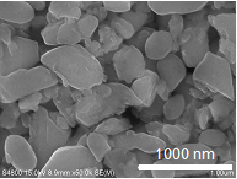 |
| FTL-100  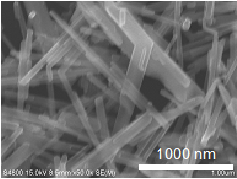 |  |  |

Fig. S1. Representative scanning electron microscope images (S-4800, Hitachi High-Technologies Co., Japan) of the seven forms of TiO_2_ used in the study.

| AMT-100  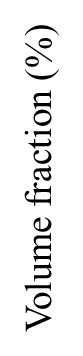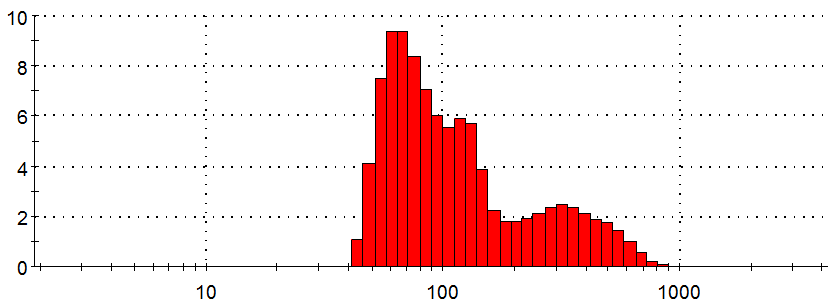  Particle size (nm) | MT-150AW  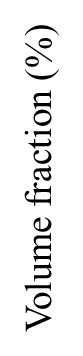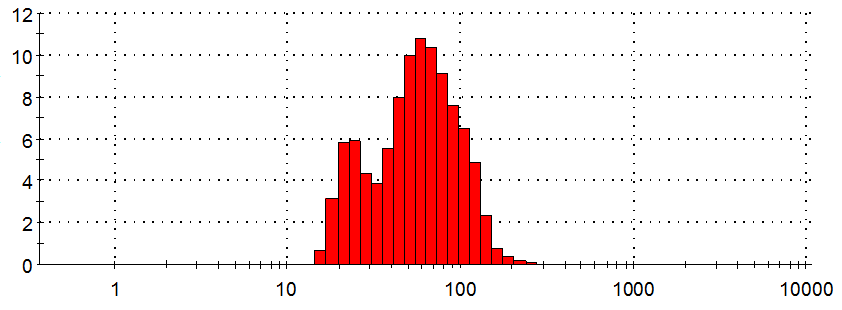Particle size (nm) |
| --- | --- |
| TTO-S-3  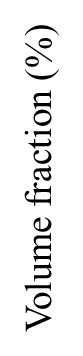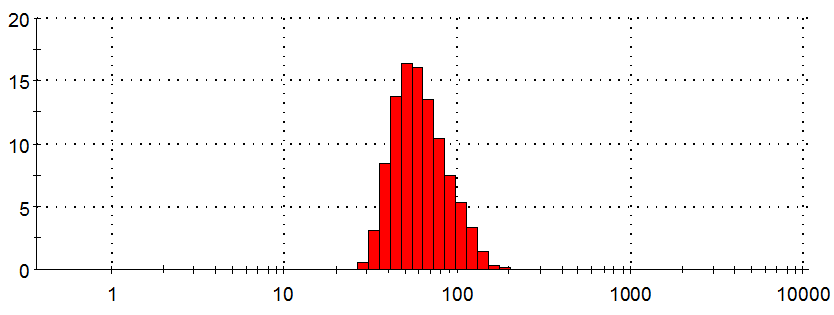  Particle size (nm) | TTO-S-3 (Coated)  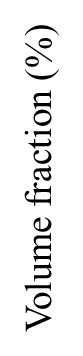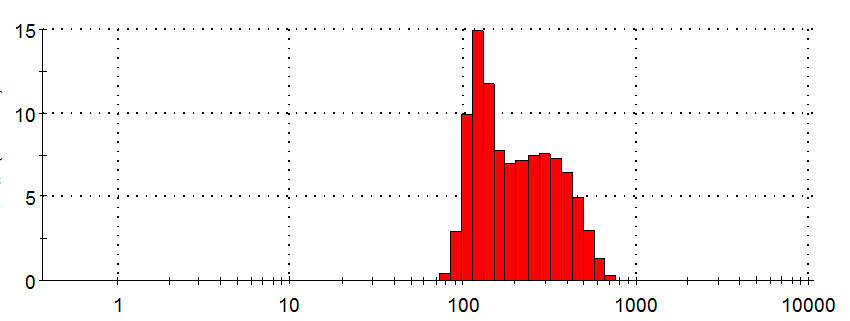Particle size (nm) |
| P25  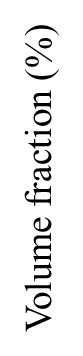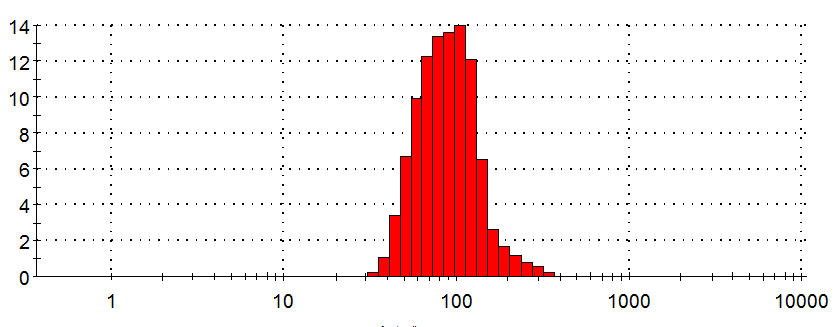Particle size (nm) | MP-100  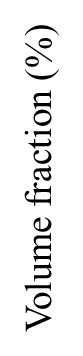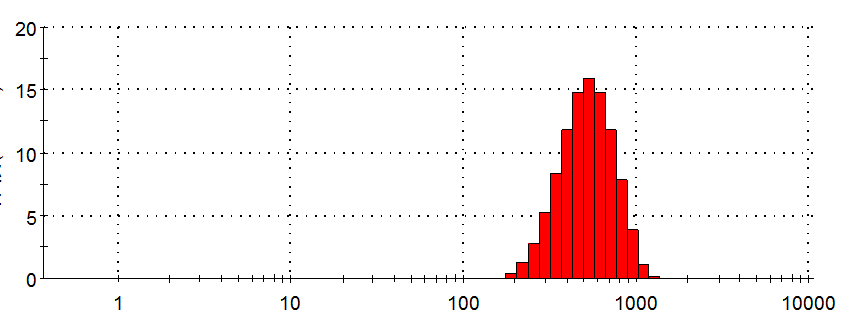Particle size (nm) |

Fig. S2-1. Volume-based size distributions of the TiO_2_ formulations, as measured by means of dynamic light scattering (Zetasizer Nano ZS; Malvern Instruments Ltd., UK).

| AMT-100  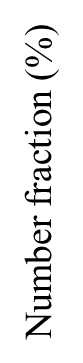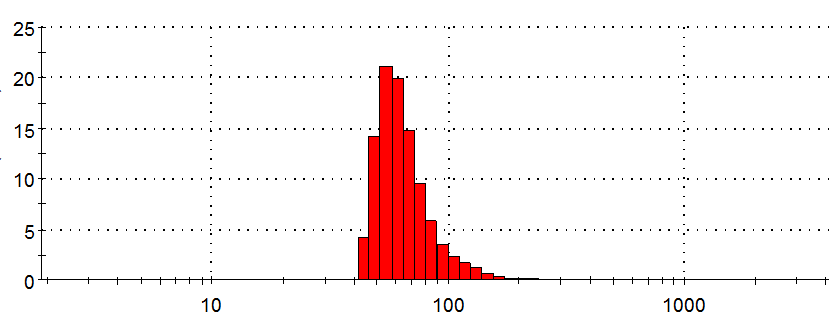  Particle size (nm) | MT-150AW  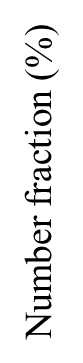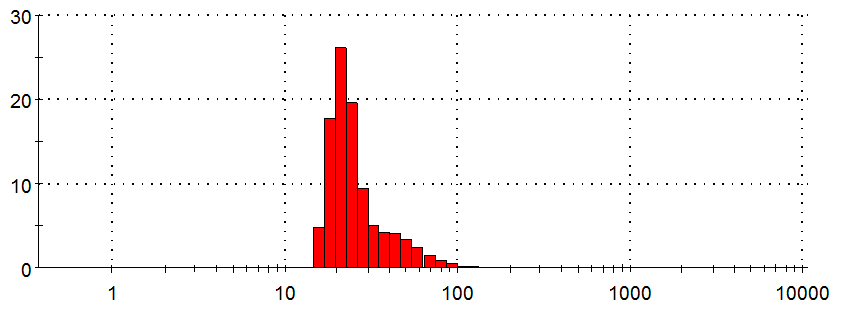Particle size (nm) |
| --- | --- |
| TTO-S-3  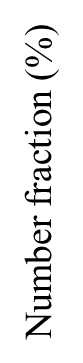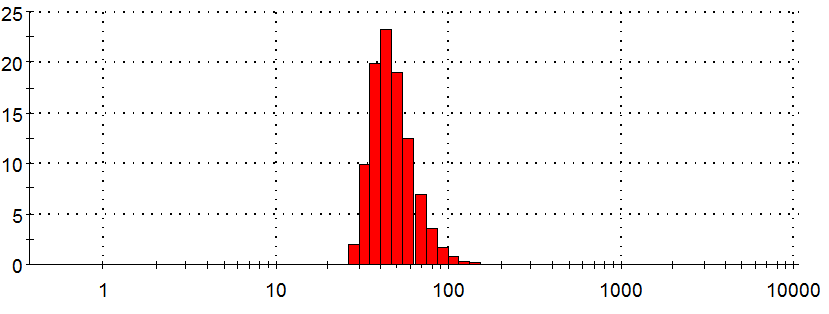  Particle size (nm) | TTO-S-3 (Coated)  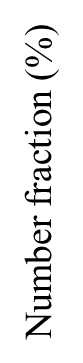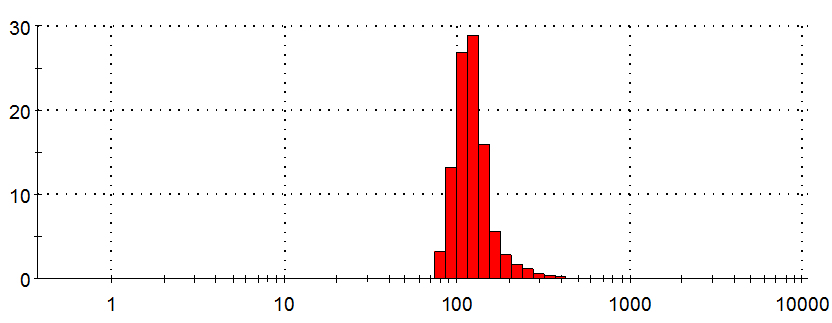Particle size (nm) |
| P25  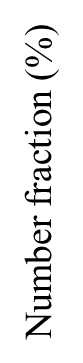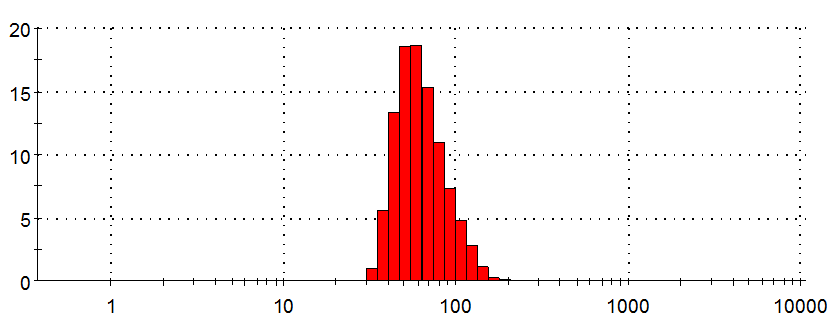Particle size (nm) | MP-100  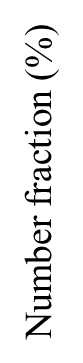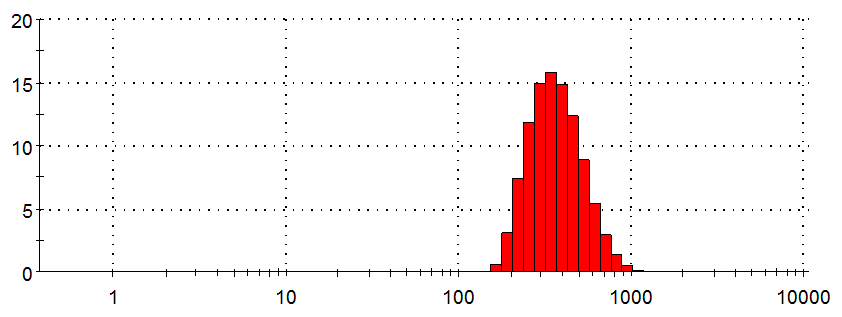Particle size (nm) |

Fig. S2-2. Number-based size distributions of the TiO_2_ formulations, as measured by means of dynamic light scattering (Zetasizer Nano ZS; Malvern Instruments Ltd., UK).


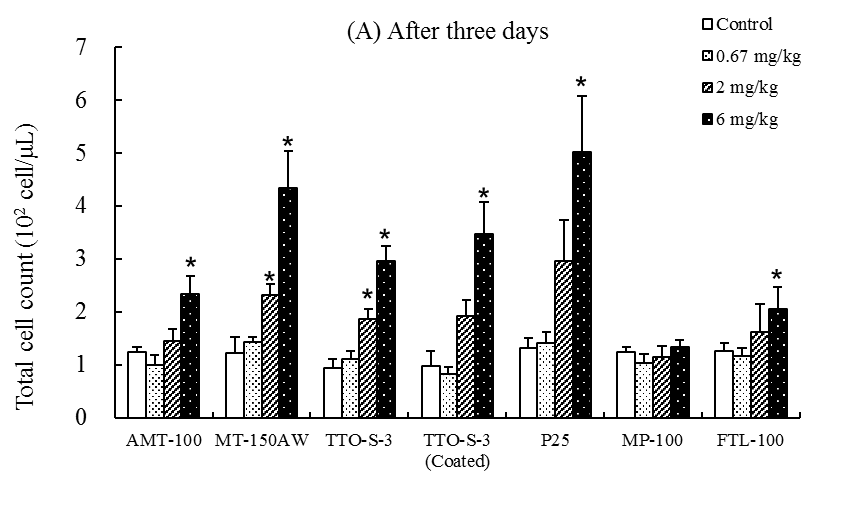


1. At three days


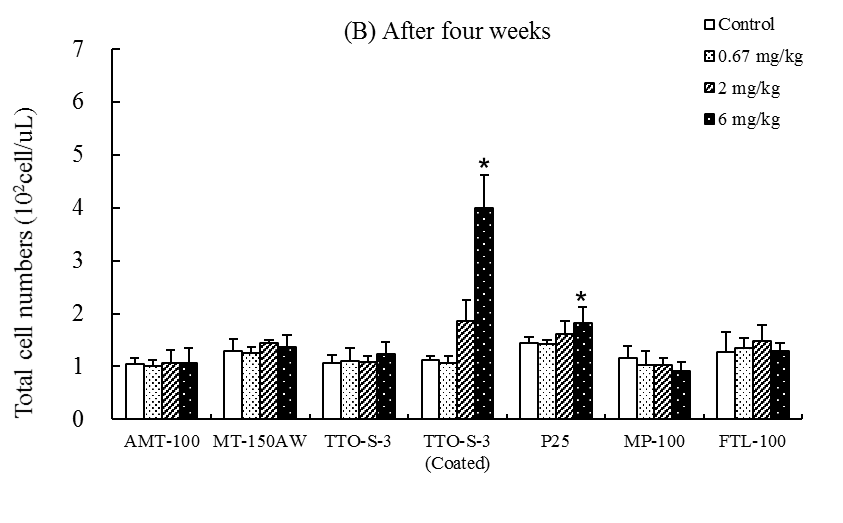


1. At four weeks


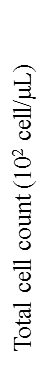


Fig. S3. Total cell count in bronchoalveolar lavage fluid at three days (A) and at four weeks (B) after intratracheal administration of various forms of TiO_2_. Values are presented as average ± SD. * significant difference from control group (*P* < 0.05).


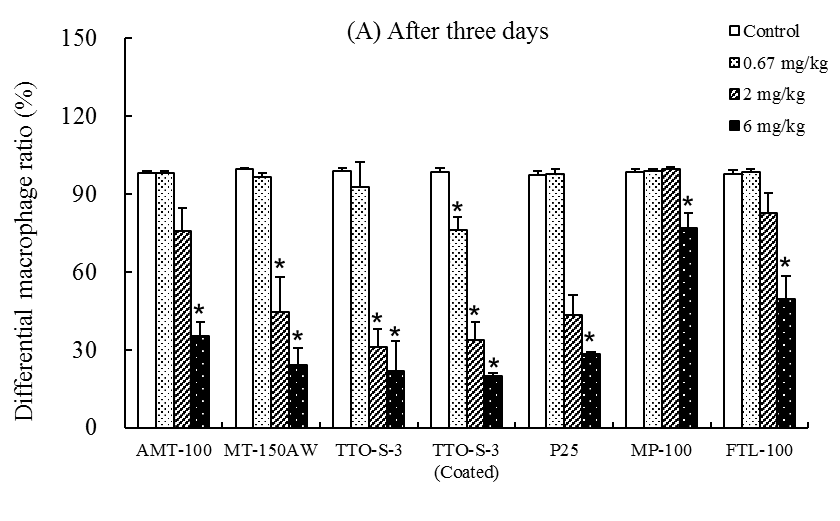


1. At three days


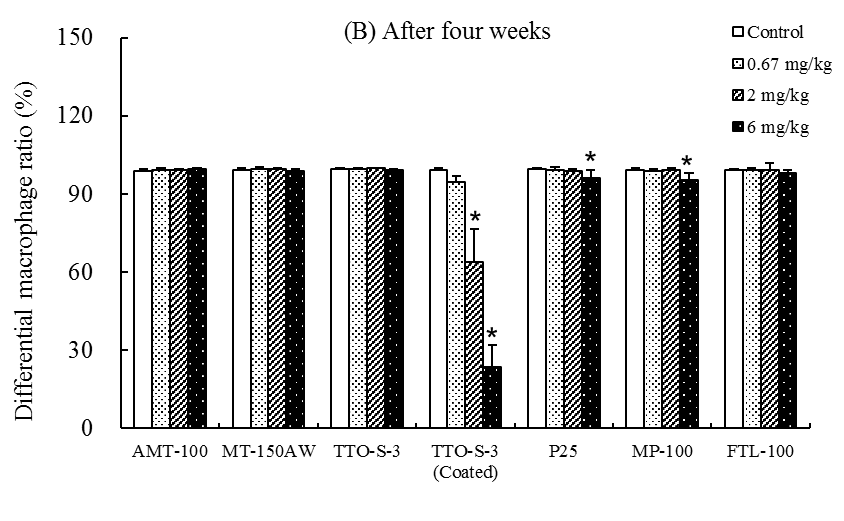


1. At four weeks

Fig. S4. Differential macrophage ratio in bronchoalveolar lavage fluid at three days (A) and at four weeks (B) after intratracheal administration of various forms of TiO_2_. Values are presented as average ± SD. * significant difference from control (*P* < 0.05).


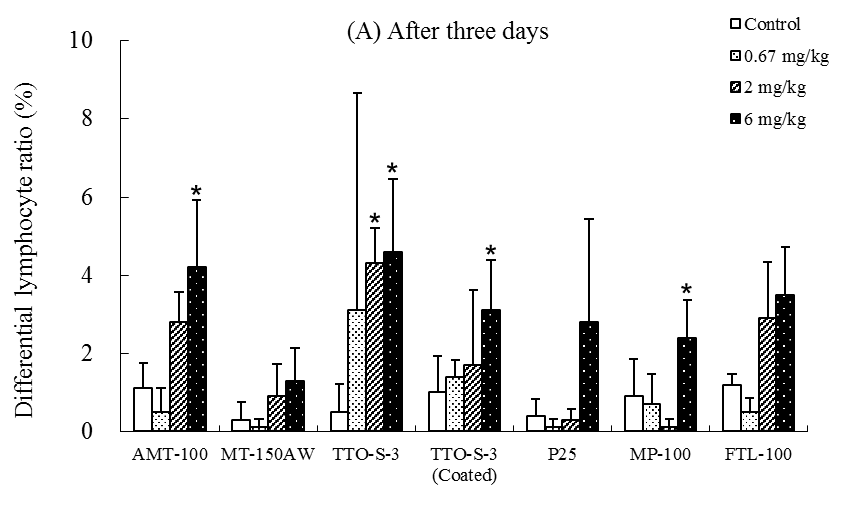


1. At three days


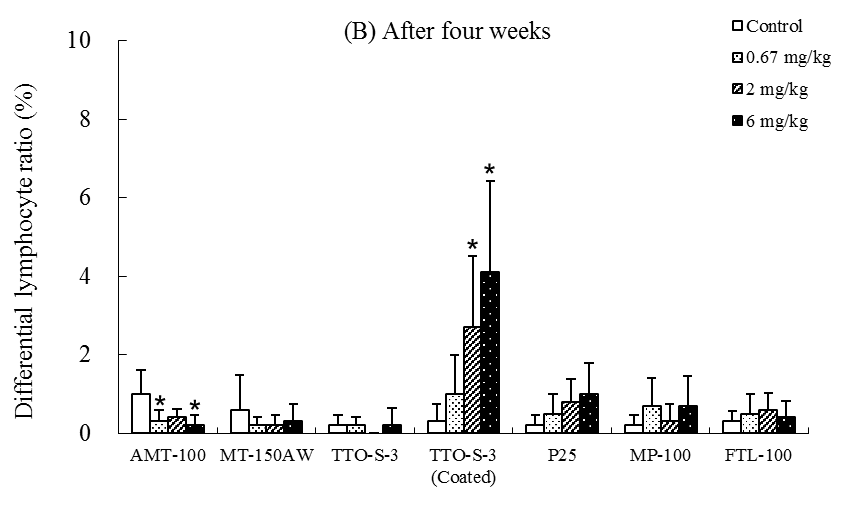


1. At four weeks

Fig. S5. Differential lymphocyte ratio in bronchoalveolar lavage fluid at three days (A) and at four weeks (B) after intratracheal administration of various forms of TiO_2_. Values are presented as average ± SD. * significant difference from control (*P* < 0.05).


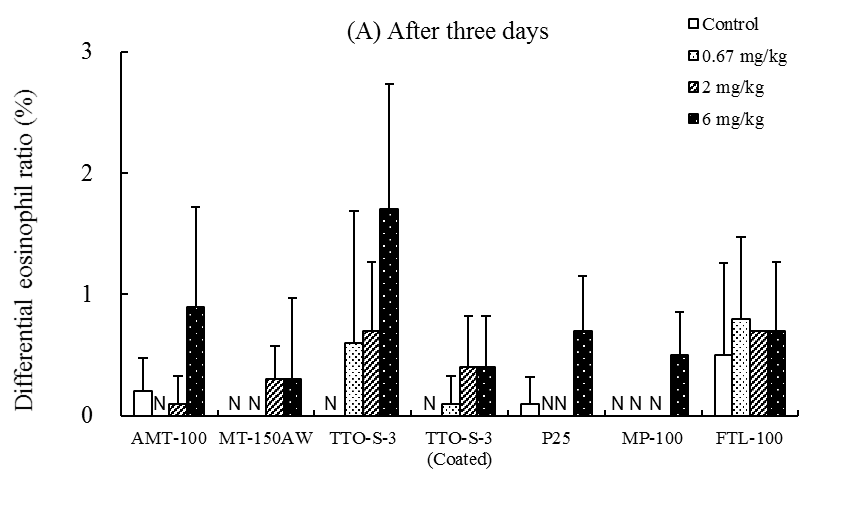


1. At three days


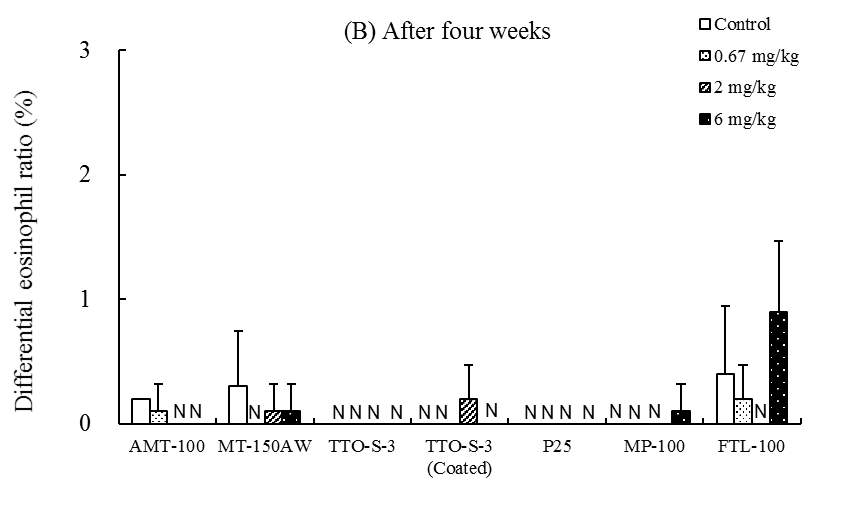


1. At four weeks

Fig. S6. Differential eosinophil ratio in bronchoalveolar lavage fluid at three days (A) and at four weeks (B) after intratracheal administration of various forms of TiO_2_. Values are presented as average ± SD. * significant difference from control (*P* < 0.05). “N” indicates no eosinophils were observed.


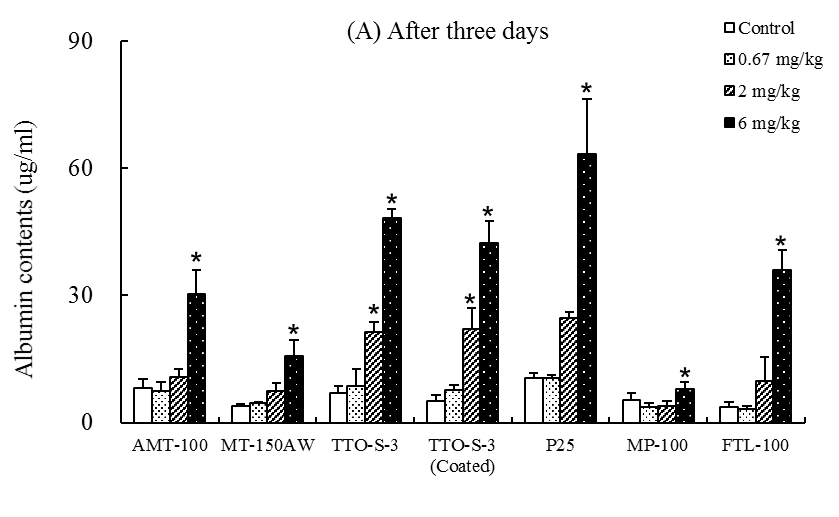


1. At three days


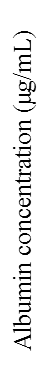


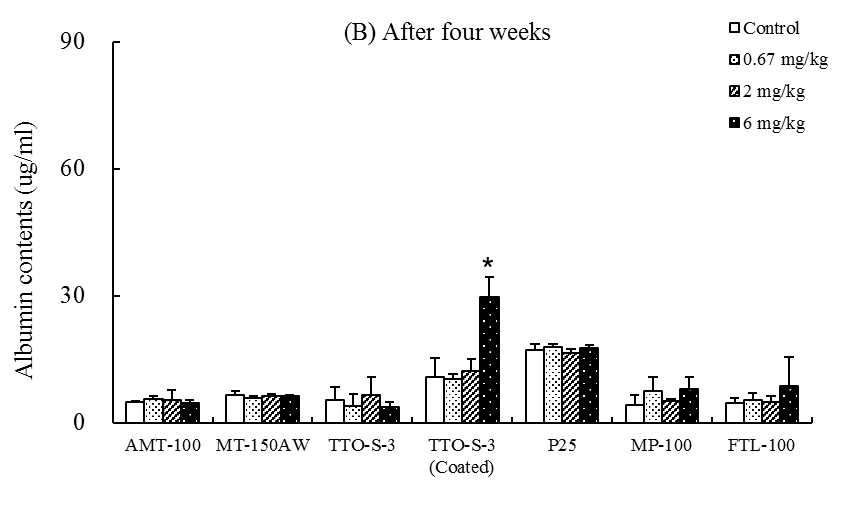

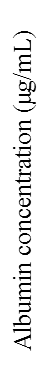


1. At four weeks

Fig. S7. Concentration of albumin in bronchoalveolar lavage fluid at three days (A) and at four weeks (B) after intratracheal administration of various forms of TiO_2_. Values are presented as average ± SD. * significant difference from control (*P* < 0.05).

**
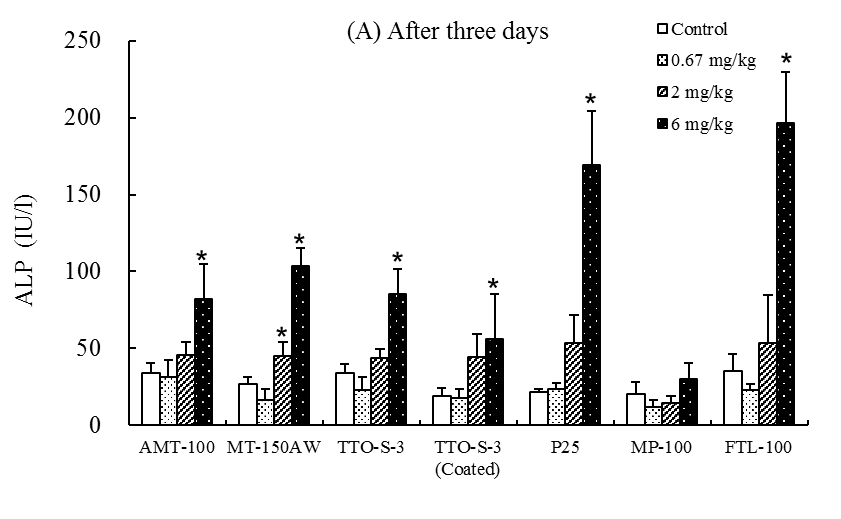
**

1. At three days


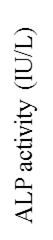


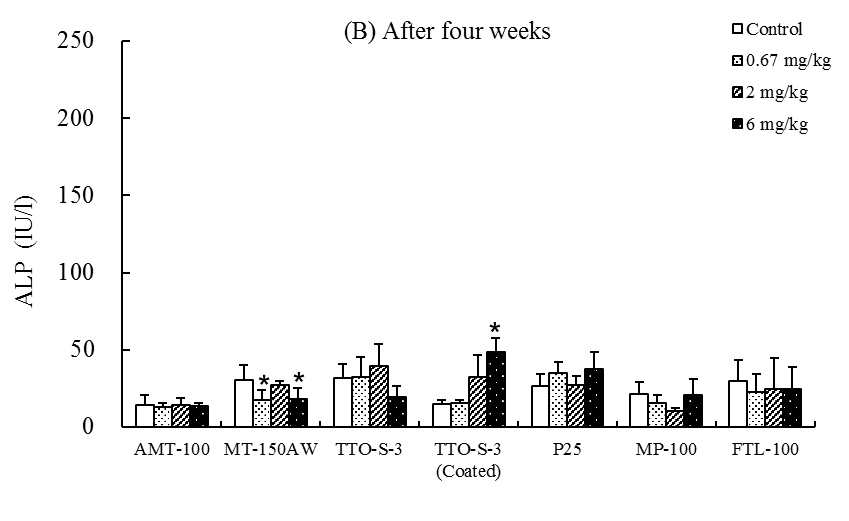


1. At four weeks


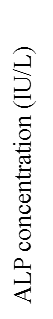


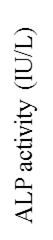


Fig. S8. Activity of alkaline phosphatase (ALP) in bronchoalveolar lavage fluid at three days (A) and at four weeks (B) after intratracheal administration of various forms of TiO_2_. Values are presented as average ± SD. * significant difference from control (*P* < 0.05).
